# National CAPS (Cryopyrin-Associated Periodic Syndrome) Registry

**DOI:** 10.1186/1546-0096-9-S1-P1

**Published:** 2011-09-14

**Authors:** C Bibalo, G Paloni, L Lepore, M Gattorno, M Finetti, F Zulian, G Martini, M Alessio, M Cattalin, A Stabile, D Rigante, A Insalaco, R Manna, R Gallizzi, L Obici, L Cantarini, S Martino, R Cionsolini

**Affiliations:** 1 IRCCS Burlo Garofolo, Trieste, Italy; 2 IRCCS Gaslini, Genova; 3 Policlinico Giustiniani, Padova, Italy; 4 Ospedale Federico II, Napoli, Italy; 5 Policlinico Spedali Civili, Brescia, Italy; 6 Università Cattolica Sacro Cuore-Roma, Italy; 7 Ospedale Pediatrico Bambin Gesù – Roma, Italy; 8 Università Cattolica Sacro Cuore, Roma, Italy; 9 Policlinico G. Martino, Messina, Italy; 10 IRCCS San Matteo, Pavia, Italy; 11 Policlinico Le Scotte, Siena, Italy; 12 Ospedale Infantile Regina Margherita S.Anna,Torino,Italy; 13 Ospedale Santa Chiara, Pisa, Italy

## Introduction

The aim of the present study was to evaluate the long term follow-up of patients enrolled in the Italian Registry of cryopirin associated periodic sindrome (CAPS).

## Patients and methods

The Italian CAPS Registry started in 2004 and has currently enrolled 29 patients: 16 with Chronic Infantile Neurologic Cutaneous Articular Syndrome (CINCAs), 8 with Muckles Welles syndrome (MWS) and 5 with Familial Cold Urticaria (FCU). 16 patients were treated with Anakinra (IL-1 receptor antagonist) at 1 mg/kg/day. The Child Health Questionnaire (CHQ-PF 50) was used to assess the health related quality of life.

##  Results

Rapid clinical and laboratory improvement was observed in all patients, but six patients who declined treatment and experienced progressive course of the disease. All the treated patients maintained persistent remission; 5 patients required an increase of the dosage to 300 mg per day. No adverse effects were recorded except injection site reactions.Until today, 8 of the 16 patients initially treated with Anakinra have subsequently switched to Canakinumab (monoclonal human antiIL-1β antibody) at the initial dose of 150 mg every 8 weeks.

## Conclusion

IL-1 inhibition seems to be crucial in modifying the natural history of CAPS. Long-term follow up of patients treated with Anakinra established its efficacy and tolerability. The same evaluation will be done for Canakinumab, that appears to be equally effective but whose side effects still need to be evaluated. The bimonthly administration of Canakinumab may be certainly useful, especially in pediatric population, however its long half-life implies a tight surveilance in case of infections.

